# Supplementary material for: Sleep Quality and Duration in Children That Consume Caffeine: Impact of Dose and Genetic Variation in ADORA2A and CYP1A
Source: Genes (Basel). 2023 Jan 22;14(2):289. doi: 10.3390/genes14020289 (PMC9956387; doi:10.3390/genes14020289)
Supplement: Supplementary file 1 [file genes-14-00289-s001.zip › genes-2076830-supplementary.pdf]

Sleep quality and duration in children that consume caffeine: Impact of dose and genetic variation in *ADORA2A* and *CYP1A*

SUPPLEMENTARY MATERIALS

Table S1. Standardized caffeine content by type of beverage

Category	Standard conversion	Caffeine (mg)	Source
Coffee	8oz Starbucks Medium Roast Coffee	155	https://globalassets.starbucks.com/assets/94fbcc2ab1e24359850fa1870fc988bc.pdf
Espresso drink	USDA 1oz Espresso Shot	64	https://fdc.nal.usda.gov/
Tea	USDA 8oz Ready to drink Black Tea	47	https://fdc.nal.usda.gov/
Soda	12oz Pepsi	38	https://www.pepsicobeveragefacts.com/
Energy drink	8oz Redbull	75.7	https://www.redbull.com/ca-en/energydrink/red-bull-energy-drink-ingredients-list

Table S2. Concordance between self-reported ethnicity and genomic-derived ancestry

	Genomic derived ancestry, %				
**Self-report ethnicity**	**EUR (n = 3835)**	**AFR (n=914)**	**EAS (n=60)**	**AMR (n=52)**	**Admixed (n=810)**
White	76.7	0.0	0.0	1.9	0.6
Black	0.3	91.5	0.0	0.0	5.9
Asian	0.4	0.0	80.0	0.0	2.6
Hispanic	15.5	1.5	1.7	96.2	63.0
Other	7.1	7.0	18.3	1.9	27.9

Table S3. Binomial logistic regression models assessing the impact of ADORA2A and CYP1A genotypes on sleep duration and quality

	Recommended Sleep Duration			Disturbed Sleep Quality		
	**OR[1]**	**95% CI**	**p**	**OR[1]**	**95% CI**	**p**
**ADORA2A model**						
rs5751876						
T/T	reference			reference		
C/T	1.01	0.86 - 1.19	0.891	1.01	0.85 - 1.22	0.851
C/C	1.00	0.84 - 1.20	0.988	1.09	0.90 - 1.34	0.352
Caffeine intake (mg/kg/day)	0.85	0.72 - 1.00	0.055	1.09	0.93 - 1.28	0.282
rs5751876 x Caffeine	0.96	0.76 - 1.20	0.694	0.86	0.68 - 1.01	0.233
Gender						
Girl	reference			reference		
Boy	1.05	0.94 - 1.17	0.375	1.01	0.89 - 1.13	0.875
Concomitant Psychotropic Use						
No	reference			reference		
Yes	0.73	0.57 – 0.93	0.011	1.89	1.49 – 2.42	$1.9x10^{-7}$
**CYP1A model**						
rs2472297						
C/C	reference			reference		
C/T	1.00	0.87 - 1.16	0.989	1.03	0.90 - 1.19	0.665
T/T	1.07	0.76 - 1.52	0.692	0.93	0.66 - 1.33	0.725
Caffeine intake (mg/kg/day)	0.81	0.73 - 0.90	8.1×10^{-5}	1.13	1.02 - 1.25	0.018
rs2472297 x Caffeine	1.06	0.71 - 1.58	0.772	0.76	0.45 – 1.25	0.285
Gender						
Girl	reference			reference		
Boy	1.05	0.94 - 1.17	0.379	1.01	0.90 - 1.14	0.855
Concomitant Psychotropic Use						
No	reference			reference		
Yes	0.73	0.57 – 0.93	0.011	1.91	1.50 – 2.43	$1.3x10^{-7}$
**CYP1A2 model**						
rs762551						
C/C (*1/*1)	reference			reference		
C/A or A/A (*1/*1F or *1F/*1F)	0.92	0.74 -1.14	0.461	1.12	0.89 – 1.41	0.306
Caffeine intake (mg/kg/day)	0.80	0.57 – 0.93	$6.9x10^{-7}$	1.08	0.99 – 1.18	0.084
rs2472297 x Caffeine	1.21	0.91 – 1.60	0.183	0.99	0.74 – 1.31	0.949
Gender						
Girl	reference			reference		
Boy	1.05	0.94 -1.17	0.370	1.01	0.89 – 1.14	0.881
Concomitant Psychotropic Use						

No	reference			reference		
Yes	0.72	0.56 - 0.93	0.011	1.91	1.51 – 2.43	$1.4x10^{-7}$

1 adjusted for caffeine intake, concomitant psychotropic use, gender, and population stratification by including the first 10 principal components

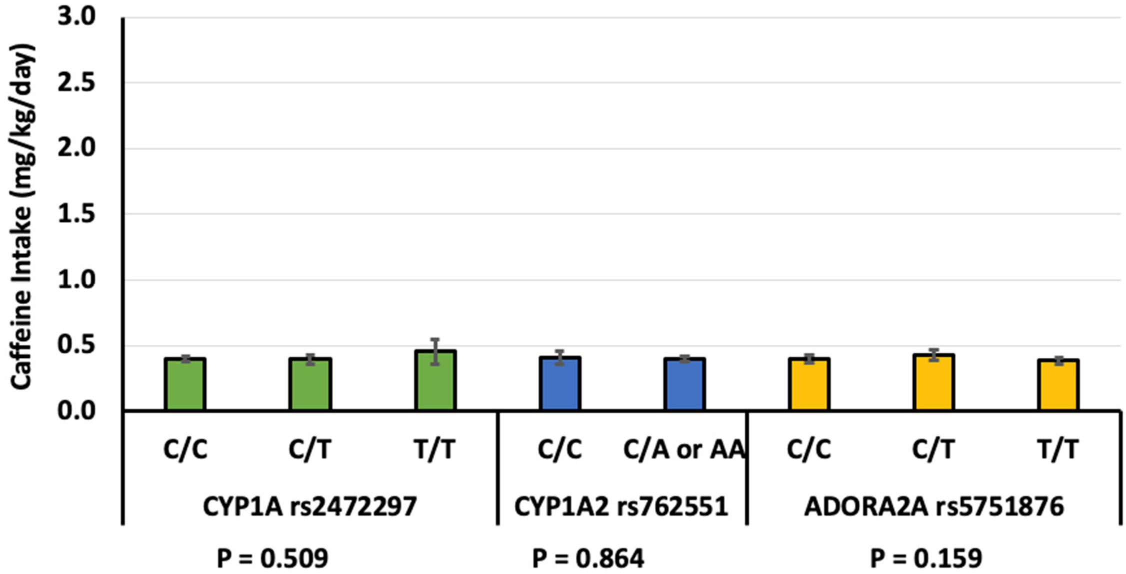

Figure S1. Mean caffeine intake by *CYP1A* and *ADORA2A* genotype adjusted for psychotropic use (i.e., SSRIs, antipsychotics, stimulants), gender and population stratification (principal components 1-10). Error bars represent 95% confidence intervals.
